# DTAF Dye Concentrations Commonly Used to Measure Microscale Deformations in Biological Tissues Alter Tissue Mechanics

**DOI:** 10.1371/journal.pone.0099588

**Published:** 2014-06-10

**Authors:** Spencer E. Szczesny, Rachel S. Edelstein, Dawn M. Elliott

**Affiliations:** 1 Department of Bioengineering, University of Pennsylvania, Philadelphia, Pennsylvania, United States of America; 2 Department of Biomedical Engineering, University of Delaware, Newark, Delaware, United States of America; Dalhousie University, Canada

## Abstract

Identification of the deformation mechanisms and specific components underlying the mechanical function of biological tissues requires mechanical testing at multiple levels within the tissue hierarchical structure. Dichlorotriazinylaminofluorescein (DTAF) is a fluorescent dye that is used to visualize microscale deformations of the extracellular matrix in soft collagenous tissues. However, the DTAF concentrations commonly employed in previous multiscale experiments (≥2000 µg/ml) may alter tissue mechanics. The objective of this study was to determine whether DTAF affects tendon fascicle mechanics and if a concentration threshold exists below which any observed effects are negligible. This information is valuable for guiding the continued use of this fluorescent dye in future experiments and for interpreting the results of previous work. Incremental strain testing demonstrated that high DTAF concentrations (≥100 µg/ml) increase the quasi-static modulus and yield strength of rat tail tendon fascicles while reducing their viscoelastic behavior. Subsequent multiscale testing and modeling suggests that these effects are due to a stiffening of the collagen fibrils and strengthening of the interfibrillar matrix. Despite these changes in tissue behavior, the fundamental deformation mechanisms underlying fascicle mechanics appear to remain intact, which suggests that conclusions from previous multiscale investigations of strain transfer are still valid. The effects of lower DTAF concentrations (≤10 µg/ml) on tendon mechanics were substantially smaller and potentially negligible; nevertheless, no concentration was found that did not at least slightly alter the tissue behavior. Therefore, future studies should either reduce DTAF concentrations as much as possible or use other dyes/techniques for measuring microscale deformations.

## Introduction

A primary area of interest in biomechanics is the study of structure-function relationships within tissues and biomaterials. Given the hierarchical organization of most biological tissues, investigation of these relationships requires examination of the tissue structure and mechanical behavior across multiple length scales. To this end, various experimental techniques (e.g. optical confocal microscopy, X-ray diffraction, Raman and infrared spectroscopy, atomic force microscopy) have been used over the past several decades to measure micro- and nanoscale deformations within numerous tissues, including bone, tendon, cartilage, annulus fibrosus of the intervertebral disc, meniscus, and cardiovascular tissues [1]–[9]. This information is vital not only for understanding the mechanisms underlying normal tissue function, but also for identifying the structural causes of mechanical impairment with disease and developing novel biomaterials that mimic native tissue function. Additionally, measuring the *in situ* mechanical strains at the cellular level can identify the mechanical stimuli that are transmitted to cells through their immediate local environment [10]–[14]. These observations can help determine how mechanical cues affect cell behavior and tissue remodeling, which is essential for successfully regenerating diseased tissue or growing tissue engineered replacements.

**Figure 1:**
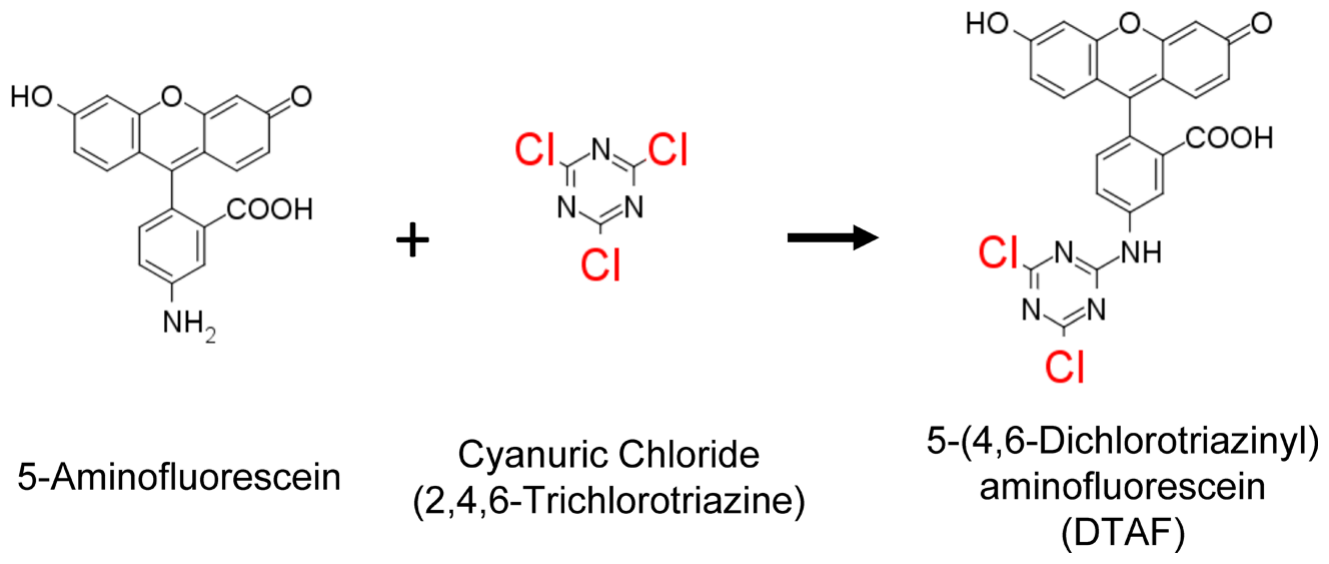
DTAF synthesis and structure. DTAF is synthesized by conjugating aminofluorescein with cyanuric chloride. Extracellular matrix proteins can then be fluorescently labeled through the reaction between the remaining chloro groups (highlighted in red) attached to the triazine ring and free amine groups on the protein.

Fluorescent labeling of the extracellular matrix with dichlorotriazinylaminofluorescein (DTAF) is commonly used to visualize microscale deformations in soft collagenous tissues with confocal microscopy [8]–[10], [15]–[23]. DTAF is synthesized through the reaction of aminofluorescein with cyanuric chloride (trichlorotriazine) (Fig. 1) [24]. Under basic conditions, DTAF binds to proteins in the extracellular matrix through reactions between the chloro groups in the triazine ring and free amine groups found on lysine side-chains and protein N-termini [24], [25]. With the extracellular matrix fluorescently labeled, microscale tissue deformations can be measured by multiple techniques, including texture correlation [10], [17], [26] and tracking the displacements of photobleached lines [8], [9], [20], [22]. Although most experiments have used relatively high concentrations of DTAF (≥2000 µg/ml) [9], [10], [15]–[22], only one study reported conducting pilot testing to determine the effects of DTAF at these concentrations on tissue mechanics [20]. While they found no significant influence of DTAF staining, preliminary testing in our lab has suggested that DTAF may substantially alter the mechanical behavior of tendon.

The objective of this study was to determine whether high DTAF concentrations alter tendon fascicle mechanics and if a concentration threshold exists below which any observed effects are negligible. Incremental strain testing was performed to evaluate changes in fascicle quasi-static and viscoelastic properties with DTAF staining. Additionally, multiscale experimental testing and modeling of fascicles stained at a low DTAF concentration were compared with previous results at 2000 µg/ml [22] to identify the mechanisms responsible for the changes in fascicle mechanics. Finally, we performed constant strain rate testing to determine the influence of the loading protocol on our results. This information is valuable for guiding the continued use of this fluorescent stain in future multiscale experiments and for interpreting the results of previous work.

## Methods

**Figure 2:**
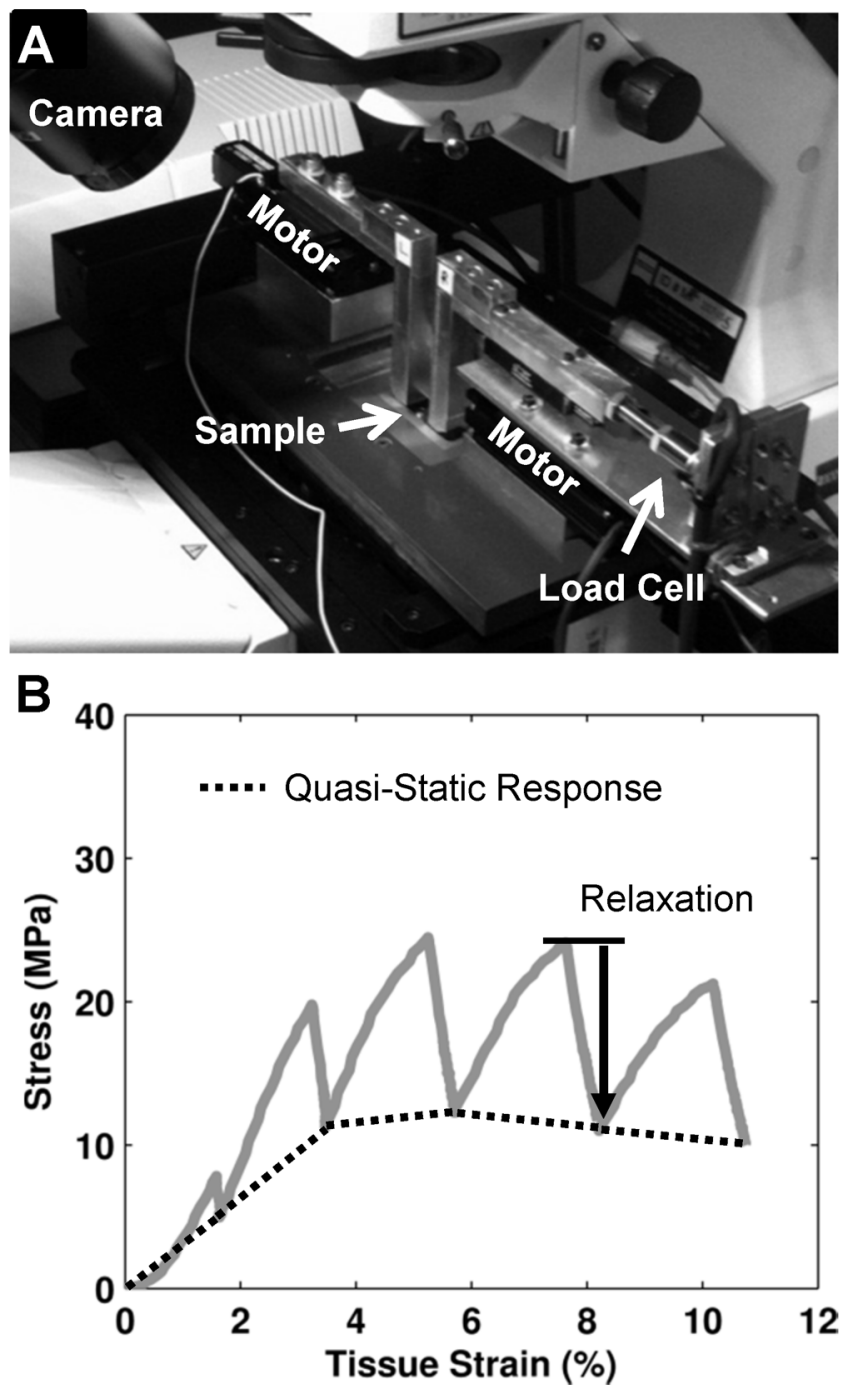
Experimental setup and quantification of tissue macroscale response. (A) Uniaxial tension device mounted on a confocal microscope. (B) Representative plot of tissue mechanical response highlighting portions used to quantify the macroscale tensile properties (i.e. quasi-static tensile modulus and incremental percent stress relaxation).

A total of 28 tendon fascicles were gently teased with tweezers from the tails of eleven 7–8 month-old Sprague-Dawley rats sacrificed for a separate study. Collection of the tails was approved by the Institutional Animal Care and Use Committee of the University of Pennsylvania (Protocol Number: 804776). Rat tail tendon fascicles are an accepted model for multiscale investigations of tendon [2], [12], [18], [19], [27]–[30] since they have a simplified and aligned structure yet contain the basic collagenous hierarchical organization common to most tendons [31], [32] and can be individually extracted intact without cutting, and potentially damaging, the tissue [32]. Stained and non-stained samples were tested in a bath of phosphate buffered saline (PBS) attached to a uniaxial tensile device mounted on an inverted confocal microscope (LSM 510 META/5 LIVE; 25X LD LCI Plan-Apochromat lens; Zeiss) (Fig. 2A). Prior to testing, the stained samples were incubated for 20 min at room temperature in a 0.1 M sodium bicarbonate buffer (pH 9.0) with a specified concentration of 5-(4,6-dichlorotriazinyl)aminofluorescein (DTAF; Life Technologies). The non-stained samples were incubated under the same conditions in the buffer without DTAF. After staining, both samples were washed with copious amounts of PBS and marked with india ink ±5 mm from their midpoint. Tissue strains were measured optically by tracking the positions of the ink marks with a macroscopic camera (scout scA1400-17gm; Basler). The samples were gripped between sheets of sandpaper within aluminum clamps and placed in the PBS bath above the microscope. To measure the tissue cross-sectional area, the ends of each sample were rotated by 360° immediately before testing. Images of the sample profile using reflected light (633 nm) were obtained with the microscope at the points along the sample length where the major axis was in a horizontal or vertical orientation. These images were used to determine the major and minor sample lengths, and the cross-sectional area was calculated assuming the samples were elliptical (0.177±0.047 mm^2^). The samples were then rotated back into a straight orientation and tested as described below.

### Incremental Strain Testing

Twenty fascicles were cut into two adjacent 35 mm proximal and distal sections with one section stained with DTAF and the other serving as a paired non-stained control. Four pairs of samples were tested at five DTAF concentration levels: 0, 2.5, 10, 100, and 2000 µg/ml (concentrations of 2000 µg/ml and higher are typically used in multiscale experiments [9], [10], [17]–[22]). An equal number of tests were performed with the distal and proximal portions of each fascicle used as the stained sample. The 0 µg/ml samples were used to determine any baseline differences between the distal and proximal portions of a fascicle.

An incremental strain testing protocol, similar to previous multiscale mechanical studies [22], was used to evaluate the impact of DTAF on fascicle quasi-static and viscoelastic properties. Briefly, a 1 mN preload (∼6 kPa) was applied to the tissue to define the reference length (20.6±0.2 mm). The sample was preconditioned by applying five cycles of 2% grip-to-grip strain at 1%/sec and then allowed to recover at the reference length for 10 min. Incremental displacements were applied to 2, 4, 6, 8, and 10% grip-to-grip strains at 1%/sec followed by a 15 min relaxation period. As mentioned above, tissue strains were determined by the displacements of ink marks applied to the sample surface. The mechanical behavior was quantified by calculating the quasi-static tensile modulus and incremental percent relaxation at each applied grip-to-grip strain value (Fig. 2B). The effect of DTAF was determined by computing the difference in the quasi-static moduli and stress relaxation between the stained and non-stained paired samples. For the 0 µg/ml sample set, paired differences in mechanics were calculated as proximal values minus distal values and (for percent difference in stress relaxation) normalized by distal values. Parametric t-tests were used to determine if the paired differences were different from zero with statistical significance set at p<0.05 and trends at p<0.10.

### Multiscale Testing and Modeling

The microstructural mechanisms for the observed effects of DTAF were investigated by multiscale testing and modeling of tendon fascicles [22]. Briefly, four fascicles (45 mm long) were stained with a 10 µg/ml DTAF solution. Each fascicle was preloaded (reference length: 30.6±0.3 mm), preconditioned, and allowed to recover as described above. After the recovery period, a set of four lines, each 2 µm wide and separated by 100 µm, were photobleached through the tissue depth in 5 µm increments with a laser diode (489 nm, 100 mW) at three locations along the fascicle length: the sample center and ±5 mm from the center. Testing consisted of incremental displacements to 2, 4, 6, and 8% grip-to-grip strains at 1%/s followed by a 15 min relaxation. Microscale image stacks (15 fps; 0.53×0.53×1.24 µm/pixel) were taken of the photobleached lines before testing and at the end of each relaxation period [22]. Note 15 min was sufficient to reduce the rate of stress relaxation so that microscale measurements could be obtained at a quasi-static state (i.e., the load dropped less than 5% during the 3 min image capture period).

The microscale tissue deformations were determined by converting the image stacks obtained at each applied strain level into single composite images of the photobleached lines spanning the entire sample width [22]. Fibril strains were defined as the change in the distance between line pairs compared to their positions at 0% applied strain. Fibril:tissue strain ratios were calculated as the average fibril strain divided by the macroscale tissue strain determined from the ink mark displacements. Microscale shear strains were measured as the angle made between each line and the direction perpendicular to the fascicle axis. The overall level of interfibrillar sliding was quantified by the standard deviation of the measured shear strains. Finally, the fibril:tissue strain ratios and interfibrillar sliding were averaged across the three microscale imaging locations and compared with previous data collected from rat tail fascicles stained at 2000 µg/ml [22].

A shear lag model was used to investigate whether the same physical mechanisms are responsible for the multiscale mechanics of fascicles stained at different DTAF concentrations. Previously, we found that a shear lag model, which assumes that load is transferred between discontinuous fibrils through a perfectly plastic interfibrillar shear stress, can fully explain the mechanics of fascicles stained at 2000 µg/ml [22]. Therefore, this shear lag model was used to determine if the multiscale response of the current 10 µg/ml samples could be explained by the same mechanical phenomena. The model parameters (fibril modulus, interfibrillar shear stress, mean uncrimping stretch, and standard deviation of uncrimping) were determined by fitting the average quasi-static macroscopic mechanical behavior. An upper-bound of 25 mm was used for the fibril length given the sample reference length. The model parameters were then used to predict the average fibril:tissue strain ratios. Since the fibril:tissue strain ratios were not used for calculating the model parameters, this prediction tests whether the physical mechanisms embedded in the model accurately represent the fascicle multiscale mechanics. The model performance and parameters were compared with the previous results of fascicles stained at 2000 µg/ml. Statistically significant differences were defined as values for the 10 µg/ml samples that were more than two standard deviations away from the average 2000 µg/ml values.

### Constant Strain Rate Testing

Constant strain rate ramps to failure were also preformed to determine the influence of the loading protocol and compare our results with previous findings [20]. Four sample pairs were prepared identically as described for the incremental loading protocol; however, only a concentration of 2000 µg/ml was investigated. The samples were preloaded (reference length: 20.6±0.1 mm) and preconditioned as before, but instead of applying incremental strains, the samples were tested to failure at a constant rate of 1%/sec. Macroscopic images used to calculated tissue strains were captured at 10 Hz. Tissue mechanics were quantified by curve-fitting the stress-strain behavior to determine the linear modulus [33] and calculating the ultimate tensile strength for each sample. Comparisons between stained and non-stained samples were made via paired t-tests with significance set at p<0.05.

## Results

**Figure 3:**
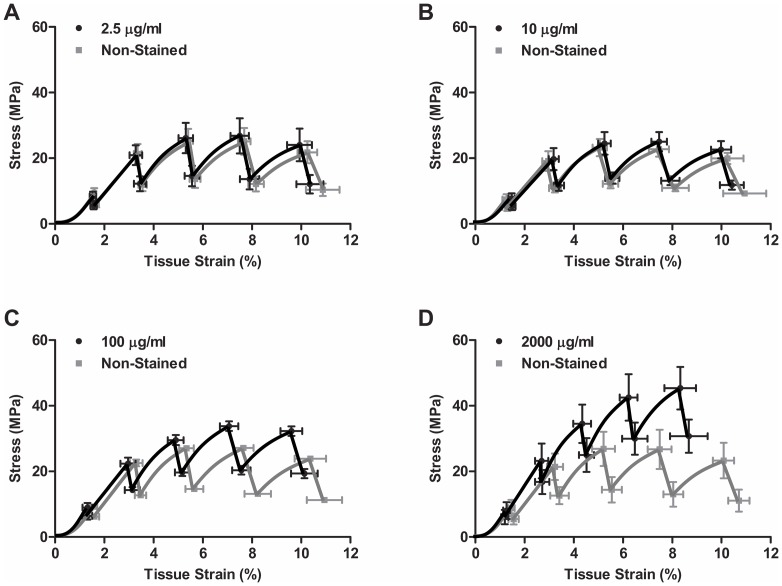
Fascicle macroscale response. Plots of the average stress-strain response to incremental loading for samples stained at (A) 2.5, (B) 10, (C) 100, and (D) 2000 µg/ml along with their paired non-stained controls.

**Figure 4:**
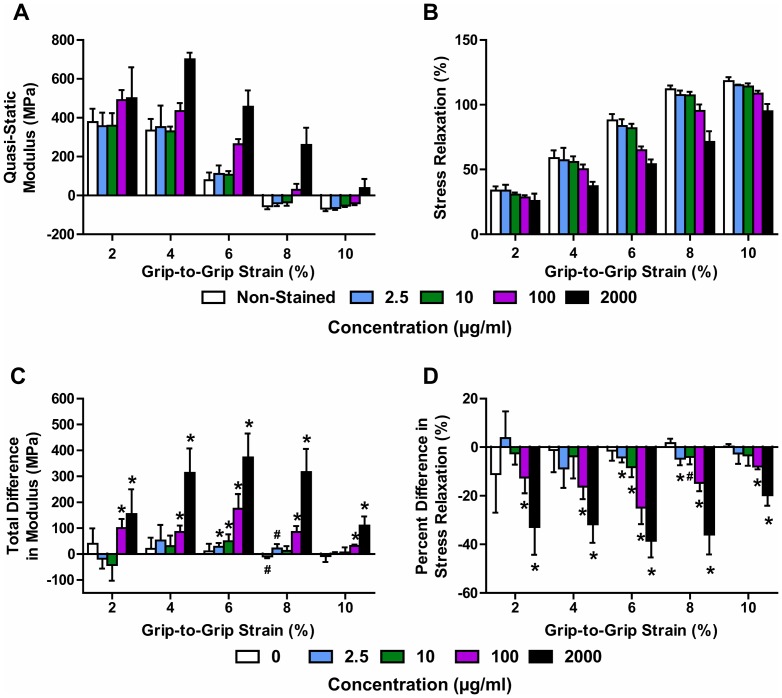
Macroscale mechanical properties as a function of DTAF concentration and applied grip-to-grip strain level. (A) Samples stained at high concentrations (>10 µg/ml) maintain large positive quasi-static moduli at greater applied grip-to-grip strains, suggesting that DTAF increases the tissue yield strain. (B) Staining also reduced the amount of stress relaxation throughout testing, with greater effects observed with increasing DTAF concentration. Note: The Non-Stained group contains all the paired non-stained control samples (n = 16). (C,D) Paired differences between stained samples and non-stained controls confirm that high DTAF concentrations produce (C) large increases in quasi-static modulus and (D) decreases in stress relaxation at all applied grip-to-grip strain levels. Lower DTAF concentrations (≤10 µg/ml) exhibited relatively small effects at 6-8% grip-to-grip stains. *p<0.05, ^#^p<0.10.

Incremental strain testing demonstrated that high concentrations of DTAF can have a substantial effect on tendon fascicle mechanics. For samples stained at the lower concentrations of 2.5 or 10 µg/ml, the average stress response was similar to their non-stained controls, with relatively minor differences observed at higher tissue strains (Fig. 3A–B). However, the 100 and 2000 µg/ml samples exhibited higher stress levels and lower tissue strains than the non-stained controls (Fig. 3C–D). Quantification of these results showed that, for the 2.5 and 10 µg/ml samples as well as the non-stained controls, the quasi-static modulus steadily decreased and became negative above 6% grip-to-grip strain (Fig. 4A). This demonstrates that the fascicles yield during testing, with a post-yield quasi-static response that is similar to perfectly plastic behavior. The 100 µg/ml samples displayed a similar response but tended to have larger moduli values that fell below zero at higher grip-to-grip strains (>8%). The quasi-static moduli for the 2000 µg/ml samples initially increased and then dropped with greater grip-to-grip strain but never became negative. These findings demonstrate that elevated DTAF concentrations increase the quasi-static tissue stiffness and delay the onset of yielding. Additionally, staining reduced the amount of stress relaxation throughout testing, with greater effects observed with increasing DTAF concentration (Fig. 4B), which shows that DTAF also reduces the viscoelastic behavior of tendon fascicles.

To more precisely evaluate the effects of DTAF staining, we directly compared the differences between the stained samples and their paired non-stained controls. Paired differences in quasi-static modulus for both the 100 and 2000 µg/ml samples were significantly different from zero at all applied grip-to-grip strains (Fig. 4C). The observed increases in modulus were greatest at 6% grip-to-grip strain, which is where the quasi-static modulus of the non-stained samples approached zero. This is consistent with DTAF increasing the tissue yield strain. Although the 2.5 and 10 µg/ml samples had similar stress responses to their non-stained controls (Fig. 3A-B), there was a small, yet statistically significant, increase in the quasi-static modulus at 6% grip-to-grip strain (Fig. 4C). This suggests that even these low concentrations of DTAF slightly delayed the point at which the fascicles began to behave in a perfectly plastic manner. The effect on stress relaxation was similar, with large decreases observed for the 100 and 2000 µg/ml samples at all strain levels and small decreases observed for 2.5 and 10 µg/ml at 6 and 8% grip-to-grip strain (Fig. 4D). These data demonstrate that DTAF alters fascicle mechanical behavior even at low concentrations levels. No statistically significant differences were found between the proximal and distal portions of fascicles from the 0 µg/ml group for either the quasi-static modulus or stress relaxation.

**Figure 5:**
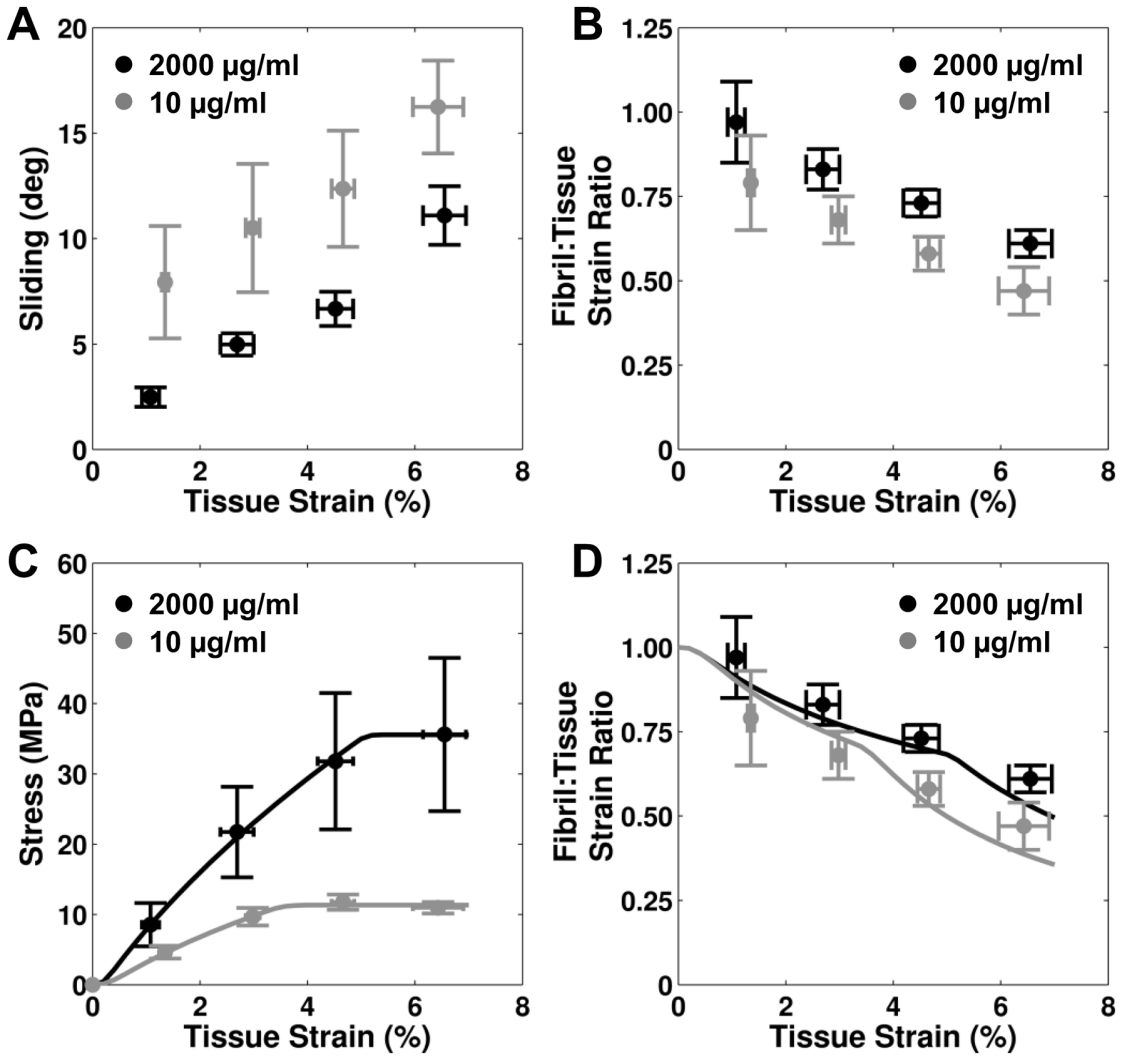
Results of multiscale testing and modeling. Multiscale testing demonstrates that higher concentrations of DTAF (A) decrease interfibrillar sliding and (B) increase fibril strains. However, the relationship between both of these microscale deformations and the macroscale tissue strains are similar between the two DTAF concentrations. Additionally, a shear lag model incorporating a perfectly plastic interfibrillar shear stress was successful in (C) fitting the macroscale fascicle mechanics (R^2^ = 0.997) and (D) predicting the microscale fibril strains (R^2^ = 0.68) of tendon fascicles stained at 10 µg/ml. These results are similar to the model performance for samples stained at 2000 µg/ml [22]. Therefore, these data suggest that while DTAF alters fascicle multiscale mechanics it doesn' change the physical mechanisms underlying fascicle behavior.

In order to identify possible causes for the effects of DTAF on fascicle macroscale mechanics, the results of multiscale testing and modeling for 10 µg/ml samples were compared to previous analysis of samples stained at 2000 µg/ml [22]. For both concentrations, the interfibrillar sliding increased and the fibril:tissue strain ratio decreased with applied tissue strain (Fig. 5A–B). However, DTAF concentrations of 2000 µg/ml produced less interfibrillar sliding and a higher fibril:tissue strain ratio compared to 10 µg/ml. This suggests that DTAF increases the load transfer between fibrils, thereby preventing the relative sliding between fibrils and increasing fibrillar strains. Additionally, a shear lag model based on a perfectly plastic interfibrillar shear stress successfully fit the average fascicle macroscale mechanics and predicted the average fibril:tissue strain ratios (Fig. 5C–D). While the performance of the model was similar for the two DTAF concentrations, the values for the fibril modulus and interfibrillar shear stress were higher for the 2000 µg/ml samples (Table 1). This suggests that the differences observed in the quasi-static macroscale mechanics with DTAF may be due to both stiffening of the individual fibrils and increasing load transfer between fibrils by strengthening the interfibrillar matrix.

**Table 1:** Parameters and Performance of Shear Lag Model.

	2000 µg/ml [22]	10 µg/ml^a^
**Parameter Values**		
Fibril Modulus (MPa)	1600±400	740*
Interfibrillar Shear (kPa)	0.35±0.11	0.11*
Mean Uncrimping Stretch	1.002±0.001	1.003
Standard Deviation of Uncrimping	0.001±0.001	0.002
**Performance**		
R^2^ of Macroscale Fit	0.996±0.003	0.997
R^2^ of Microscale Prediction	0.74±0.18	0.68

a Single values reported since model was applied to average multiscale response.

*Values fall outside two standard deviations of 2000 µg/ml data.

**Figure 6:**
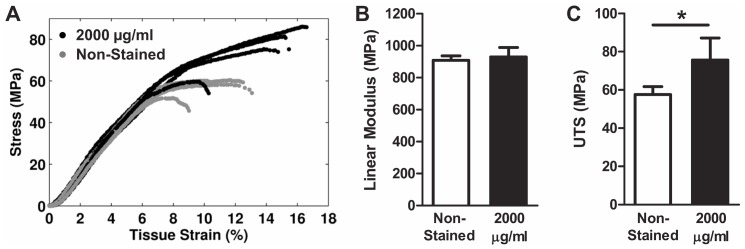
Results of constant strain rate testing. (A) Plots of the stress-strain response to constant strain rate testing for 2000 µg/ml samples and non-stained controls. (B) While the linear modulus was unaffected by DTAF staining, (C) an increase was observed for the ultimate tensile strength (UTS). *p<0.05.

To evaluate the influence of the loading protocol on these results, 2000 µg/ml samples were tested to failure under a constant strain rate. Pilot testing by Thorpe et al. of equine tendon fascicles suggested that this concentration of DTAF had no effect on fascicle mechanics for this type of loading [20]. Interestingly, we also found no difference in linear modulus between the paired stained and non-stained samples when loaded at a constant strain rate (Fig. 6). However, there was a significant increase in ultimate tensile strength with staining. This suggests that testing at a constant strain rate partly masks the effects of DTAF observed with incremental loading.

## Discussion

This study investigated whether DTAF, a fluorescent dye commonly used in multiscale testing of tissue mechanics, alters the mechanical behavior of tendon fascicles. Using an incremental loading protocol, we found that high concentrations (≥100 µg/ml) substantially affect tissue behavior by increasing the quasi-static modulus and decreasing the stress relaxation (Fig. 4). These effects are most prominent around 6% strain, which is when the quasi-static stress-strain response plateaus and the fascicles begin to behave in a perfectly plastic manner (Fig. 3), suggesting that these concentrations of DTAF delay the onset of yielding. This is concerning since the majority of previous multiscale studies of numerous biological tissues have used DTAF concentrations of 2000 µg/ml or more [9], [10], [15]–[22]. Although lower DTAF concentrations (≤10 µg/ml) also slightly alter tissue macroscale mechanics (Fig. 4), the magnitude of these effects is substantially smaller than those produced by higher concentrations and the stress-strain curves are very similar to non-stained controls (Fig. 3). Depending on the objectives and design of future studies, these effects may be negligible; therefore, DTAF concentrations up to 10 µg/ml may potentially be used to measure microscale deformations without appreciably altering tissue mechanics.

The observed effects of DTAF on the macroscale tissue behavior agree with changes in the microscale deformations. Multiscale testing demonstrated that fascicles stained at 10 µg/ml exhibited greater interfibrillar sliding and lower fibril:tissue strain ratios than samples stained at 2000 µg/ml [22] (Fig. 5A–B). Additionally, comparing the parameters predicted by the shear lag model (Table 1), DTAF appears to increase the magnitude of the interfibrillar shear stress, potentially by strengthening the interfibrillar matrix. This is consistent with the changes observed in the fibril:tissue strain ratio and interfibrillar sliding with DTAF concentration, since a higher interfibrillar shear stress would increase the transmission of strain into the fibrils and reduce interfibrillar sliding. Since the model assumes that the fascicle post-yield behavior is produced by plasticity at the fibril-matrix interface [22], an increased interfibrillar shear stress would also explain the delayed yielding and increased ultimate tensile strength observed in the macroscale fascicle mechanics. The modeling results additionally suggest that DTAF increases the fibril modulus; combined with the reduced interfibrillar sliding and greater fibril strains, this also helps explain the higher quasi-static tissue modulus for 2000 µg/ml. In fact, the lower fibril modulus for the 10 µg/ml fascicles is in better agreement with previous measurements for fibrils in native tissue [34]–[40]. Finally, given evidence that interfibrillar sliding is partly viscous in nature [2], [5], , the reduced interfibrillar sliding in the 2000 µg/ml samples is consistent with the decrease in stress relaxation observed with DTAF staining (Fig. 4B,D).

Despite the changes produced by DTAF staining in tissue multiscale behavior, the basic physical mechanisms underlying tendon fascicle mechanics are not altered. For both 10 and 2000 µg/ml, interfibrillar sliding increased while the fibril:tissue strain ratio decreased with greater applied tissue strains (Fig. 5A–B). This suggests that tendon fibrils are discontinuous and that the macroscale tissue strains result from a combination of fibril elongation and relative sliding, with interfibrillar sliding becoming more important at higher strains. Therefore, this fundamental deformation mechanism was unaffected by DTAF concentration even though the magnitudes of the fibril strains and interfibrillar sliding were different. Furthermore, the shear lag model was similarly successful in replicating the fascicle multiscale mechanics for both concentrations (Fig. 5C–D). Therefore, it appears that DTAF does not change the fundamental mechanisms underlying tendon macroscale mechanics but rather alters the mechanical properties of the individual tissue components. This suggests that the previous conclusions regarding microscale deformations and attenuation or amplification of strain transfer ratios in tissues stained with high concentrations of DTAF [9], [10], [16]–[22] are still valid but that the magnitudes of these effects may not represent native non-stained tissue.

**Figure 7:**
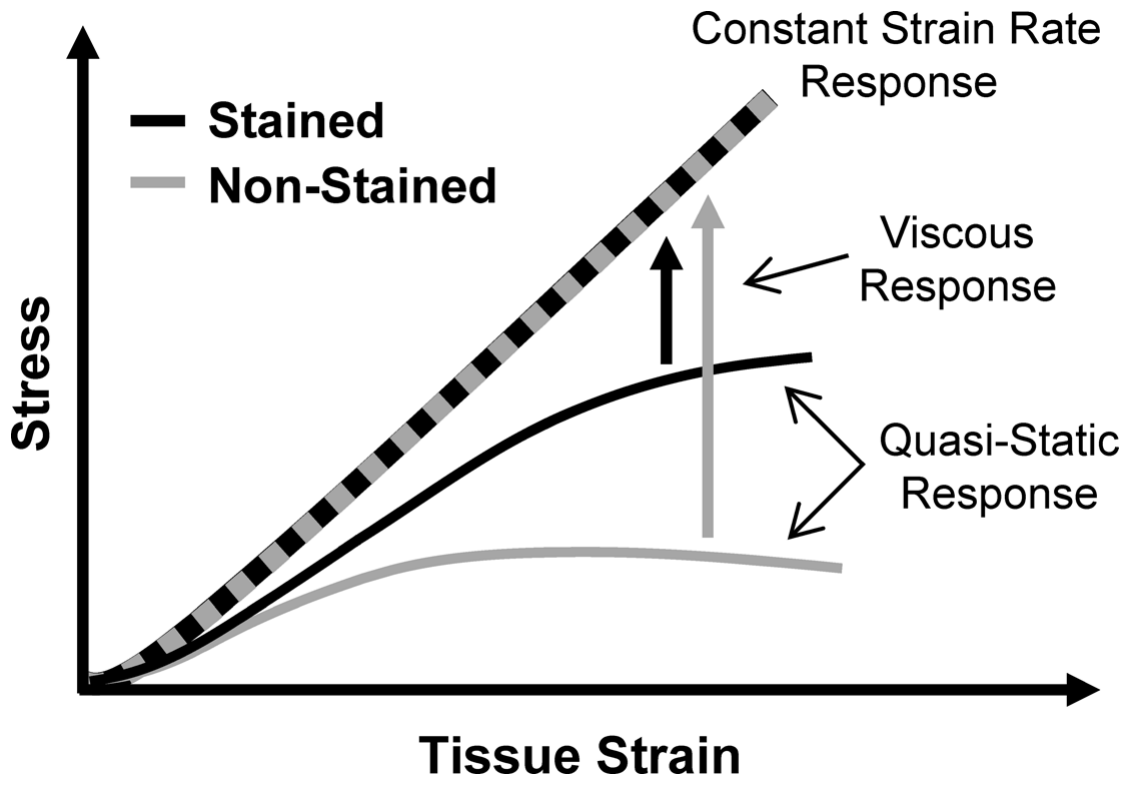
Explanation for how effects of DTAF may be masked during constant strain rate testing. Although the non-stained fascicles have a lower quasi-static modulus, they have a greater viscous response than the stained samples. Therefore, the non-stained samples stiffen more in response to the higher strain rate during the constant strain rate testing, possibly causing the stress-strain curves to overlap.

In contrast to our findings, Thorpe et al. has reported that their pilot testing of equine fascicles stained at 2000 µg/ml had no effect on fascicle mechanics [20]. While differences between rat tail and equine fascicles may explain the disagreement between our results, Thorpe et al. tested samples at a constant strain rate rather than the incremental strain protocol often used for multiscale experimentation [10]–[12], [19]–[22], [27], [28], [43]. Interestingly, we also found that the linear modulus of rat tail fascicles was not affected by DTAF staining when tested at a constant strain rate (Fig. 6). This may be due to the specific combination of effects produced by DTAF; while the quasi-static modulus increases with staining, the stress relaxation decreases and the tissue becomes less viscous. Therefore, native non-stained fascicles, which have a lower quasi-static modulus, will stiffen more than those stained with DTAF during constant strain rate testing, thereby concealing the influence of DTAF on tissue mechanics (Fig. 7). While additional testing would be required to confirm this explanation, the difference in tissue behavior between the incremental and constant strain rate testing demonstrates that the apparent effect of DTAF on tendon mechanics depends on the testing protocol.

The structural modifications produced by DTAF leading to the observed changes in tendon multiscale mechanics are unknown. It is possible that DTAF could form short crosslinks within or between collagen molecules through the reaction of both chloro groups on the triazine ring (Fig. 1). However, after the initial binding of the first chloro group, the reactivity of the remaining chloro group with amines is low. For example, all three chloro groups in cyanuric chloride, which is combined with aminofluorescein to make DTAF (Fig. 1), react with aqueous ammonia only at elevated pressures and temperatures [25]. In addition, fluorescent labeling of antibodies with DTAF shows no evidence of crosslinking between separate immunoglobulin molecules [24]. Alternatively, DTAF can react with hydroxyl groups and has been shown to bind to proteoglycans in tissue [15], [24], [25], [44]. This suggests that DTAF could link separate glycosaminoglycan chains with each other or with collagen molecules. Finally, fluorescent dyes are known to self-aggregrate and interact through non-specific binding and hydrophobic interactions [45]. In fact, we have generally found that lower DTAF concentrations (≤100 µg/ml) actually produced greater signal intensities during imaging, likely due to dye self-quenching at higher concentrations. Elevated levels of DTAF conjugation have also been found to precipitate proteins out of solution, possibly due to reversible self-aggregation [24]. Therefore, high concentrations of DTAF may cause dye molecules to accumulate, particularly in the interfibrillar space, and produce the observed effects on tissue mechanics through similar non-specific binding between tissue components.

These findings in rat tail tendon fascicles suggest that future studies investigating the microscale deformations of collagenous tissues should limit DTAF concentrations to 10 µg/ml or less. However, it is possible that the effects of DTAF may be tissue specific; the multiscale behavior of tendon fascicles has been shown to depend on tendon function and anatomical location [20], [43], [46] with additional differences observed across other tissue types [11]. Rat tail fascicles have certain unique characteristics compared to other tendons (e.g., acid-labile collagen crosslinks) that influence their mechanical behavior [40]. Nevertheless, they have the same basic hierarchical organization as other soft collagenous tissues [31], [32], suggesting that similar effects may occur with DTAF staining of other tissues. In addition, while most investigations of microscale deformations use an incremental testing protocol [10]–[12], [19]–[22], [27], [28], [43], differences observed between incremental loading and constant strain rate testing demonstrate that the effects of DTAF may not be apparent for all testing conditions. Still, it seems prudent to reduce DTAF concentrations as much as possible given the dye's potential for altering tissue mechanics and since concentrations of 10 µg/ml or less were sufficient for measuring microscale deformations in this and previous studies [8], [23]. Despite our limited sample size, we did observe small but statistically significant effects even at the lowest concentrations (Fig. 4C,D). For many studies, these small changes in tissue mechanics may be scientifically insignificant; however, if they cannot be neglected, then other dyes, including CNA35 [26], [47] or FITC, which self-aggregates less than DTAF [24], or alternative techniques (e.g., tracking displacements of cell nuclei [1], [11], [27], exploitation of tissue autofluorescence [48]) may be used for measuring microscale deformations. In any case, this study highlights the importance of carefully choosing the proper DTAF concentration levels for the particular tissue, protocol, and hypothesis in future work.

In conclusion, our results demonstrate that DTAF concentrations commonly used in the literature (≥2000 µg/ml) can dramatically alter the mechanics of tendon fascicles. These effects appear to be due to a stiffening of the collagen fibrils and strengthening of the interfibrillar matrix; however, the basic mechanisms underlying the macroscale tissue behavior appear to remain intact. This suggests that the conclusions from previous investigations of strain transfer across physical length scales using DTAF are still valid, but that the specific strain transfer magnitudes may be different than native non-stained tissue. Lower DTAF concentrations (≤10 µg/ml) produced acceptable images for measuring microscale deformations and had potentially negligible effects on tendon mechanics. Still, no concentration was found that did not at least slightly alter the tissue behavior. Therefore, future studies should either reduce DTAF concentrations as much as possible or, if the small effects of low DTAF concentrations cannot be neglected, use other dyes/techniques for measuring microscale deformations.
